# Saroglitazar protects against cadmium-induced hepatorenal injury in rats by suppressing NF-κB and upregulating Nrf2

**DOI:** 10.3389/fphar.2026.1916439

**Published:** 2026-10-05

**Authors:** Ahmed Alanazi, Hoda E. Kafl, Dalia H. El-Kashef, Ghada M. Suddek

**Affiliations:** Department of Pharmacology and Toxicology, Faculty of Pharmacy, Mansoura University, Mansoura, Egypt

**Keywords:** apoptosis, cadmium, hepatorenal, inflammation, oxidative stress, saroglitazar

## Abstract

**Introduction:**

Cadmium (Cd) toxicity is one of the major public health problems because of its long half-life (10–30 years). Exposure to Cd can cause toxicity that affects several organs, including the liver and the kidney. Saroglitazar is highlighted in this study as a potential preventative medication against hepatic-renal damage induced by Cd in rats.

**Methods:**

Cd (3 mg/kg) was injected for five successive days to induce hepatic and renal damage. Saroglitazar (2 and 4 mg/kg/day) was given orally for five successive days concurrently with Cd.

**Results:**

Saroglitazar effectively improved hepatic and renal function and structure. In the meantime, saroglitazar mitigated the oxidative stress caused by Cd; it increased the level of reduced glutathione (GSH) and the activity of superoxide dismutase (SOD) and decreased malondialdehyde (MDA) content. Saroglitazar-treated groups showed a significant reduction in hepatic and renal nuclear factor kappa B (NF-κB), tumor necrosis factor (TNF)-α, and interleukin-6 (IL-6). Furthermore, saroglitazar pretreatment significantly increased nuclear factor erythroid 2–related factor 2 (Nrf2); furthermore, there was a marked reduction in the expression of caspase-3 in both hepatic and renal tissues.

**Conclusion:**

Collectively, saroglitazar showed a protective effect against Cd-induced hepatorenal injury in rats.

## Highlights

- Saroglitazar attenuated cadmium-induced hepatic and renal injury.
- Saroglitazar possessed antioxidant properties by restoring antioxidant/oxidant balance.
- Saroglitazar inhibited hepatic and renal inflammation by targeting the NF-κB pathway.
- Saroglitazar suppressed apoptosis in both hepatic and renal tissues.

## 1 Introduction

The world widely recognizes heavy metal pollution as one of the most significant threats to human survival (Kumar et al., 2021). Lately, there has been growth in industrial activity, resulting in extended human exposure to industrial pollutants such as cadmium (Cd) (Zamani et al., 2021). Cd is considered one of the most dangerous metals that can infiltrate the water system and contaminate food or air; furthermore, various industrial and agricultural sources produce it (Farag and Alagawany, 2023). In the human body, Cd has a biologically lengthy half-life of 10–30 years (Şimşek et al., 2023). Because of the harmful effects of Cd on genes and epigenetics, researchers are still studying bioindicators for low levels of this water pollutant (Zamani et al., 2021). Studies have demonstrated that prolonged exposure to Cd leads to diverse physiological, histological, and biochemical alterations in several organs of both humans and experimental animals, as well as slow elimination from the body. Consequently, Cd primarily accumulates in the liver, kidneys, and brain (Farag and Alagawany, 2023).

The mechanisms behind the negative consequences of Cd exposure are intricate and still not completely understood (Omar and El-Sayed, 2024). It is well known that exposure to Cd significantly contributes to the development of liver and kidney damage (El-Boshy et al., 2017). Therefore, there is an urgent need for new medicines that can effectively reduce or stop the harmful effects of Cd toxicity, such as oxidative stress, inflammation, and apoptosis (Kandemir et al., 2021). Cd can hurt the kidneys by creating the Cd–metallothionein (Cd–Mt) complex in the liver. This complex leads to oxidative stress and reaches the proximal renal tubules (Farag and Alagawany, 2023). Also, Cd induces mitochondrial degradation, resulting in decreased adenosine triphosphate (ATP) production and increased formation of reactive oxygen species (ROS) (Abdelnaby et al., 2022). ROS induce the activation of heat shock protein 70 (HSP70), nuclear factor erythroid 2-related factor 2 (Nrf2), and nuclear factor kappa B (NF-κB). These proteins play a role in regulating the antioxidant responses of cells under oxidative stress. Nrf2 and NF-κB greatly enhance cells’ antioxidant defense under stress and aid in the elimination and detoxification of external substances and their harmful byproducts (Farag and Alagawany, 2023).

Saroglitazar is a novel compound derived from aryl alkoxy propionic acid and is the first approved member of the glitazar family. Patients with type 2 diabetes mellitus (T2DM) can use this medication to treat diabetic dyslipidemia and hypertriglyceridemia. Those who do not respond well to statin therapy specifically benefit from its design (Francis et al., 2023). This is the first compound to activate both peroxisome proliferator-activated receptor (PPAR)-α and PPAR-γ, and it has completed its clinical studies and received approval for worldwide clinical use. PPAR-α/γ receptors are nuclear receptor proteins that enhance ligand-dependent transcription of target genes responsible for regulation of lipid metabolism, energy production, and inflammation (Hu et al., 2023). Prior research has shown that saroglitazar yields substantial advantages in terms of enhancing lipid and glycemic indices while maintaining a favorable safety profile (Shetty et al., 2015). Saroglitazar has shown protective effects against fluorouracil-induced hepatorenal damage (Alharbi et al., 2024), besides renoprotective effects against unilateral ureteral obstruction (UUO)-induced renal damage (Makled and El-Kashef, 2020). Thus, this study was executed to examine whether saroglitazar could have a beneficial effect against Cd-induced hepatorenal injury with a particular focus on NF-κB and Nrf2.

## 2 Materials and methods

### 2.1 Chemicals and drugs

Cd chloride was purchased as a white crystalline powder in the form of CdCl_2_ with a molar mass of 183.31 g/mol. It was purchased from Alpha Chemika Co. (Mumbai, India), and it was used to induce multiorgan injury after it was dissolved in normal saline (0.9% NaCl) to yield a concentration of 3 mg/mL for intraperitoneal injection (ip) and was prepared immediately before use. Saroglitazar was purchased as a commercial tablet formulation (Bilypsa® 4 mg); therefore, excipient effects cannot be excluded. It was purchased from Zydus BioNext Co. (India) and suspended in carboxymethylcellulose (CMC). All chemicals used were of the finest analytical grade and were purchased from El-Nasr Chemicals Co. (Abu Zaabal, Cairo, Egypt).

### 2.2 Animals

Adult male Sprague–Dawley (SD) rats, which typically weighed between 150 and 170 g, were purchased from The Egyptian Organization for Biological Products and Vaccines (VACSERA), Giza, Egypt. The rats were kept at a temperature of 25 ± 1 °C, and a 12-h light/dark cycle was maintained for the duration of the trials. Moreover, the rats were provided with unrestricted access to standard meals and water throughout the experiment. Prior to the start of the experiment, the rats underwent a 7-day acclimatization process to the surrounding environment. All animal procedures complied with the ARRIVE guidelines and the National Institutes of Health’s requirements, as well as the Mansoura University Animal Care and Use Committee’s (MU-ACUC) authorized guidelines, code number (PHARM.PhD.23.07.25).

### 2.3 Experimental layout

Following acclimation, 32 male Sprague–Dawley rats were allocated into four groups of eight using a stratified randomization method.

#### 2.3.1 Normal control group

Rats received 0.5% CMC (orally) and normal saline (0.9% NaCl) (ip) after 2 h for five consecutive days.

#### 2.3.2 Cd group

Rats received 0.5% CMC and then, after 2 h, were injected with Cd (3 mg/kg, ip) for five consecutive days. The dose was selected on the basis of previous studies reporting reproducible induction of hepatorenal injury in rats at this dose and duration (Sulayman Aboulqassim et al., 2023; Sulayman Aboulqassim et al., 2026).

#### 2.3.3 Saro 2+ Cd group

Rats received saroglitazar (2 mg/kg, orally) (Francis et al., 2023) suspended in 0.5% CMC; then, after 2 h, they were injected with Cd (3 mg/kg, ip) for five consecutive days.

#### 2.3.4 Saro 4+ Cd group

Rats received saroglitazar (4 mg/kg, orally) (Francis et al., 2023) suspended in 0.5% CMC; then, after 2 h, they were injected with Cd (3 mg/kg, ip) for five consecutive days.

On the sixth day, rats were anesthetized using secobarbital sodium (50 mg/kg, ip) and then euthanized by cervical dislocation. To separate serum, the blood was left for 10 min to clot and then centrifuged for 10 min at 1000 g (4 °C). Serum was used for the assessment of hepatic and renal biomarkers. Kidney and liver tissues were collected and homogenized in 20 mM Tris-HCl (pH 7.4) containing 1 mM EDTA and then centrifuged at 3000 g for 20 min (at 4 °C), and the supernatants were collected and stored at −80 °C for the enzyme-linked immunosorbent assay (ELISA); 10% (v/v) formalin in normal saline was used to fix the slices of the kidney and liver for 48 h prior to preparing paraffin blocks for histological and immunohistochemical (IHC) analyses.

Although eight rats were initially allocated per group, two animals in the Cd group were excluded in most analyses because of hemolysis affecting serum biochemistry and tissue autolysis compromising histopathology. No mortality occurred. Sample size was based on prior studies using similar Cd models. Inclusion/exclusion criteria were prespecified, and the primary outcome was defined as hepatic and renal injury severity; no welfare-related deaths occurred. No saroglitazar-only group was included; therefore, potential baseline effects of saroglitazar cannot be excluded.

### 2.4 Measurement of serum biomarkers

The serum levels of aspartate aminotransferase (AST) and alanine transaminase (ALT), urea, creatinine, albumin, alkaline phosphatase (ALP), gamma-glutamyl transferase (GGT), and bilirubin were measured using Spinreact kits (SPAIN) according to the instructions provided.

### 2.5 Determination of oxidative stress

The hepatic and renal contents of malondialdehyde (MDA; cat. no. MD2529, Biodiagnostic, Egypt) and the levels of reduced glutathione (GSH; cat. no. GR2511, Biodiagnostic, Egypt) were measured using a spectrophotometric assay according to the manufacturer’s protocols. Superoxide dismutase (SOD) activity was evaluated using a rat SOD ELISA kit (cat. no. CSB-E08555r, CUSABIO BIOTECH, United States) according to the instructions provided. All ELISA measurements were performed by personnel blinded to group allocation.

### 2.6 Determination of protein content

Protein content in hepatic and renal tissues was quantified using the GeNei protein determination kit (cat. no. 2624800021730, GeNei Laboratories Pvt. Ltd., India) by following the Bradford method (Bradford, 1976).

### 2.7 Determination of nuclear factor erythroid 2–related factor 2

Hepatic and renal Nrf2 levels were quantified in tissue homogenates using a rat ELISA kit (cat. no. E1083Ra, Bioassay Technology Laboratory, China) by following the manufacturer’s protocol.

### 2.8 Assessment of inflammatory mediators

The hepatic and renal levels of NF-κB (CSB-E13148r, CUSABIO BIOTECH, United States), TNF-α (CSB-E11987r, CUSABIO BIOTECH, United States), and IL-6 (R6000B, Bio-Techne Ltd, United States) were measured in tissue homogenates using commercially available ELISA kits according to the manufacturer’s guidelines.

### 2.9 Histopathology

The liver and kidney were dissected from rats and fixed in neutral buffered formalin for 24 h. The samples (n = 6) were dehydrated in ascending grades of alcohol, cleared with xylene, and embedded in paraffin wax. The embedded samples were cut with a microtome into 5 μm thick sections. The sections were stained with hematoxylin and eosin (H&E) and then examined using a light microscope (Olympus CH2, Japan). The histopathological alterations were graded using a semiquantitative scoring system on a scale of 0–3, where 0 = no lesion, 1 = mild, 2 = moderate, and 3 = severe. The parameters were evaluated and scored as follows: hepatocellular necrosis, inflammation, and steatosis for the liver and necrosis, inflammation, congestion, and fibrosis for the kidney. For each group, six independent tissue sections were examined for each parameter and scored individually (Al-Rasheedi et al., 2026).

### 2.10 Immunohistochemical analysis of caspase-3

Using normal histology techniques, liver and kidney specimens (n = 6) were embedded in paraffin and preserved in formalin. Sections that were 5 μm thick were cut, deparaffinized, rehydrated, and then put through an antigen recovery procedure; 0.1 M citrate buffer was used for antigen recovery, and it was cooked for 10 min at a high temperature in a microwave oven. To quench endogenous peroxidase activity, the sections were then submerged in 3% H_2_O_2_ in methanol for 20 min. These sections were then immersed in 10% normal goat serum for 30 min to prevent non-specific binding. The primary anti-caspase-3 antibodies (Caspase 3 Polyclonal Antibody, Thermo Fisher, cat. no. PA1-29157, dilution 1:1,000) were then placed on the slides and allowed to incubate. After washing in PBS, three times for 5 min each, the sections were coated with the secondary antibody and allowed to sit at room temperature for 30 min. Following a PBS wash, color development was achieved using 3,3′-diaminobenzidine tetrahydrochloride (Sigma) in 50 mmol Tris-HCl at pH = 7.5 for 5 min. Hematoxylin was used for counterstaining, and kidney and liver sections were viewed under a microscope using a blind method. For quantification in hepatic and renal sections, the percentage of immunopositive cells was assessed using ImageJ software (Version 1.54; NIH, United States).

### 2.11 Statistical analysis

GraphPad Prism V9.01 (Graph Software Inc., CA, United States) was used for statistical calculations. For comparison between groups, normality was assessed using the Shapiro–Wilk test and homogeneity of variance using Levene’s test. Parametric data were analyzed using one-way ANOVA followed by the Tukey–Kramer *post hoc* test. Non-parametric data (histopathology scores) were analyzed using the Kruskal–Wallis test followed by Dunn’s test. The *p*-value was fixed at <0.05.

## 3 Results

### 3.1 Impact of saroglitazar (2 and 4 mg/kg) on liver biomarkers

Injection of Cd significantly (*p* < 0.05) increased the levels of ALT and AST by 2- and 2-fold, respectively, when compared with the control group. Upon treatment with saroglitazar (2 and 4 mg/kg), there was a marked (*p* < 0.05) decrease in the levels of ALT (by 26% and 40%, respectively) and AST (by 10% for saroglitazar 4 mg/kg) when compared with the Cd group. For both ALT and AST, there was a significant difference between the Saro 4+ Cd group and the Saro 2+ Cd group. Both groups of saroglitazar are significantly (*p* < 0.05) different from the control group (Figures 1A,B).

**FIGURE 1:**
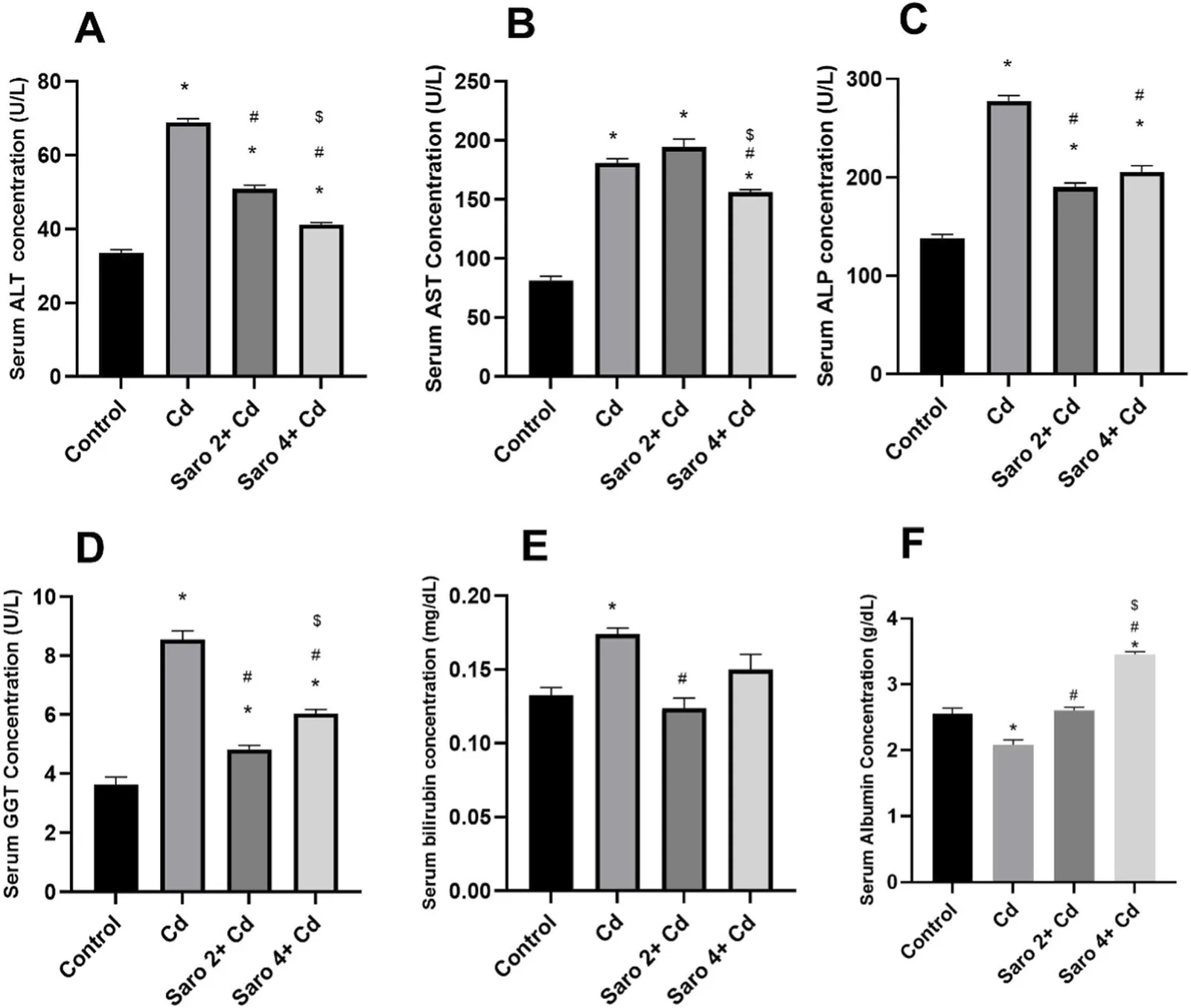
Impact of saroglitazar (2 and 4 mg/kg) on liver biomarkers in Cd-treated rats. **(A)** ALT. **(B)** AST. **(C)** ALP. **(D)** GGT. **(E)** Bilirubin. **(F)** Albumin. Values are expressed as the mean ± SE (n = 6/group). *, #, and $ significant from control, Cd, and Saro 2+ Cd groups, respectively (*p* < 0.05), using ANOVA followed by the Tukey–Kramer test. ALB, albumin; ALP, alkaline phosphatase; ALT, alanine aminotransferase; AST, aspartate transaminase; Cd, cadmium; GGT, gamma-glutamyl transferase.

Regarding ALP and GGT, Cd significantly (*p* < 0.05) increased their levels by 2- and 2-fold, respectively, compared with the control group. Oral treatment with saroglitazar (2 and 4 mg/kg) significantly (*p* < 0.05) decreased the levels of ALP (by 31.5% and 26%, respectively) and GGT (by 43.6% and 29%, respectively) when compared with the Cd group. There was still a significant (*p* < 0.05) difference between the control group and both groups of saroglitazar. For ALP, there was no significant difference between the Saro 4+ Cd group and the Saro 2+ Cd group. However, for GGT, there was a significant difference between the Saro 4+ Cd group and the Saro 2+ Cd group (Figures 1C,D).

Concerning bilirubin, Cd markedly (*p* < 0.05) increased its level by 1-fold relative to the control group. Concurrent treatment with saroglitazar (2 and 4 mg/kg) decreased the levels of bilirubin (by 28.8% and 13.7%, respectively) when compared with the Cd group. There was no significant difference between the Saro 4+ Cd group and the Saro 2+ Cd group (Figure 1E).

Figure 1F shows that injection with Cd significantly (*p* < 0.05) decreased albumin levels by 18.5% compared with the control group. Saroglitazar (2 and 4 mg/kg) markedly (*p* < 0.05) increased albumin levels by 1- and 1.6-fold, respectively, when compared with the Cd group. The Saro 4+ Cd group was significantly (*p* < 0.05) different from the Saro 2+ Cd group, and there was a significant (*p* < 0.05) difference between the control group and the Saro 4+ Cd group.

### 3.2 Impact of saroglitazar (2 and 4 mg/kg) on kidney biomarkers

Injection of Cd significantly (*p* < 0.05) increased levels of creatinine and urea by 1.5- and 2-fold, respectively, compared with the control group. Upon treatment with saroglitazar (2 and 4 mg/kg), creatinine levels were decreased (by 14% and 30%, respectively) and urea levels were reduced (by 0.4% and 20%, respectively) when compared with the Cd group. For creatinine, the Saro 4+ Cd group was significantly (*p* < 0.05) different from the Saro 2+ Cd group, and there was a significant (*p* < 0.05) difference between the control group and the Saro 2+ Cd group. For urea, the Saro 4+ Cd group was significantly (*p* < 0.05) different from the Saro 2+ Cd group, and there was a significant (*p* < 0.05) difference between the control group and both groups of saroglitazar (Figures 2A,B).

**FIGURE 2:**
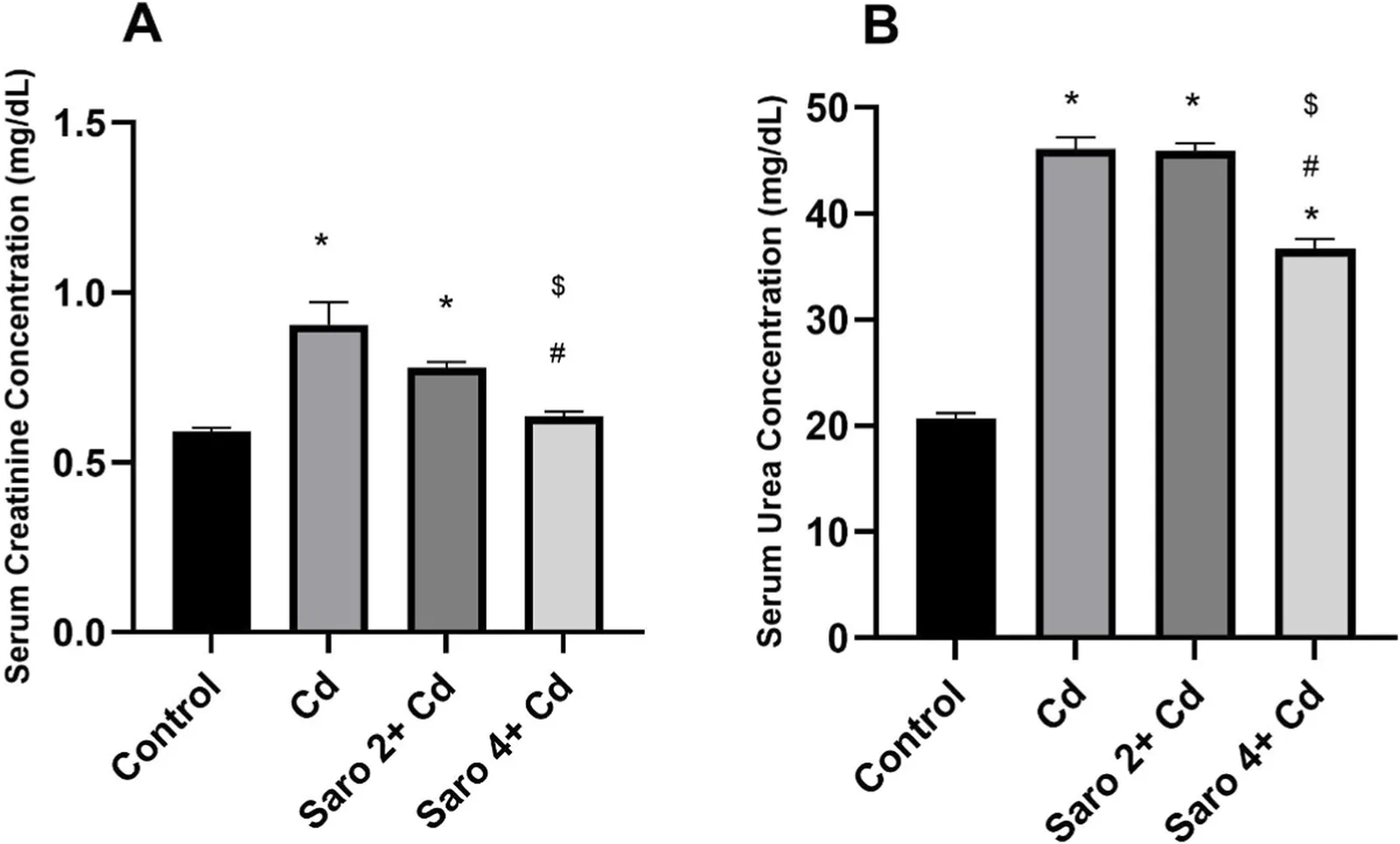
Impact of saroglitazar (2 and 4 mg/kg) on kidney biomarkers in Cd-treated rats. **(A)** Creatinine. **(B)** Urea. Values are expressed as the mean ± SE (n = 6/group). *, #, and $ significant from control, Cd, and Saro 2+ Cd groups, respectively (*p* < 0.05), using ANOVA followed by the Tukey–Kramer test. Cd, cadmium.

### 3.3 Impact of saroglitazar (2 and 4 mg/kg) on oxidant/antioxidant status

Intoxication with Cd significantly (*p* < 0.05) increased both hepatic and renal MDA contents by 13.6- and 12.9-fold compared with the control group. Upon treatment with saroglitazar (2 and 4 mg/kg), hepatic MDA contents were decreased by 20% and 81.6%, respectively, and renal MDA contents were reduced by 13% and 42.7%, respectively, when compared with the Cd group. Saro 4 mg/kg was significantly (*p* < 0.05) different from Saro 2 mg/kg. There was a significant (*p* < 0.05) difference between the control group and both groups of saroglitazar (Figure 3).

**FIGURE 3:**
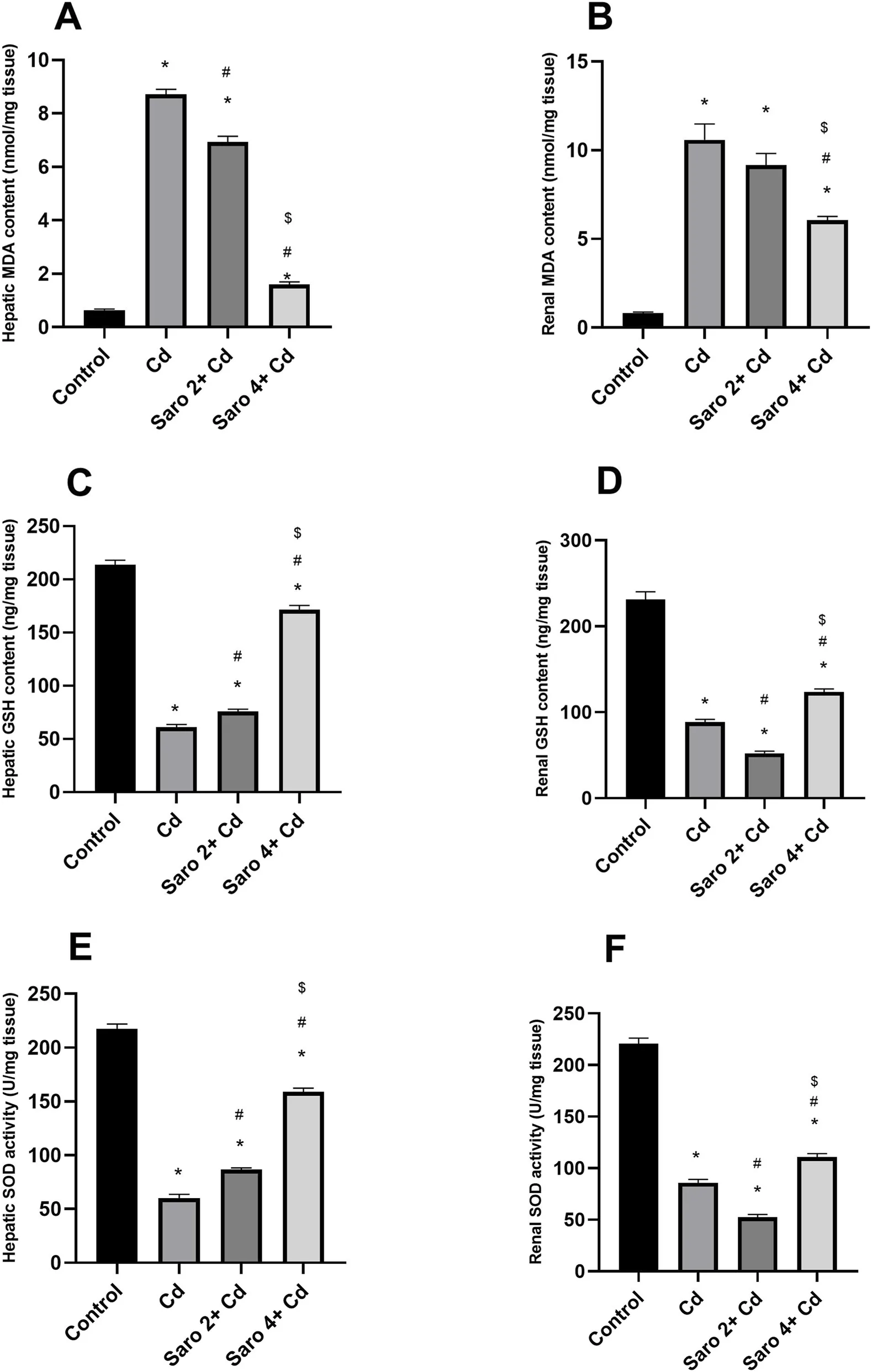
Impact of saroglitazar (2 and 4 mg/kg) on hepatic and renal oxidant/antioxidant status in Cd-treated rats. **(A)** Hepatic MDA. **(B)** Renal MDA. **(C)** Hepatic GSH. **(D)** Renal GSH. **(E)** Hepatic SOD. **(F)** Renal SOD. Values are expressed as the mean ± SE (n = 6–8/group). *, #, and $ significant from control, Cd, and Saro 2+ Cd groups, respectively (*p* < 0.05), using ANOVA followed by the Tukey–Kramer test. Cd, cadmium; GSH, reduced glutathione; MDA, malondialdehyde; SOD, superoxide dismutase.

Cd injection resulted in a significant (*p* < 0.05) decrease in hepatic GSH levels by 71% and renal GSH by 61.7% and marked reduction in hepatic SOD activity by 72% and renal SOD activity by 61% when compared with the control group. Concurrent treatment with saroglitazar (2 and 4 mg/kg) substantially (*p* < 0.05) increased hepatic GSH levels by 1-fold and 2.8-fold, respectively, and increased hepatic SOD activity by 1-fold and 2.6-fold, respectively. Oral administration of saroglitazar (4 mg/kg) increased renal GSH levels by 1.4-fold and renal SOD by 1-fold when compared with the Cd group; however, saroglitazar (2 mg/kg) failed to restore renal GSH and SOD levels. The Saro 4+ Cd group was significantly (*p* < 0.05) different from the Saro 2+ Cd group. There was a significant (*p* < 0.05) difference between the control group and both groups of saroglitazar (Figure 3).

### 3.4 Impact of saroglitazar (2 and 4 mg/kg) on Nrf2 protein levels

Cd injection significantly decreased Nrf2 hepatic levels by 94% and renal levels by 82% compared with control. Upon treatment with saroglitazar (2 and 4 mg/kg), hepatic Nrf2 protein levels were increased by 1.9-fold and 9.8-fold, respectively. Regarding renal Nrf2 protein levels, saroglitazar (4 mg/kg) increased it by 1.7-fold when compared with the Cd group; however, saroglitazar (2 mg/kg) failed to elevate Nrf2 protein levels. The Saro 4+ Cd group was significantly (*p* < 0.05) different from the Saro 2+ Cd group. There was a significant (*p* < 0.05) difference between the control group and both groups of saroglitazar (Figure 4).

**FIGURE 4:**
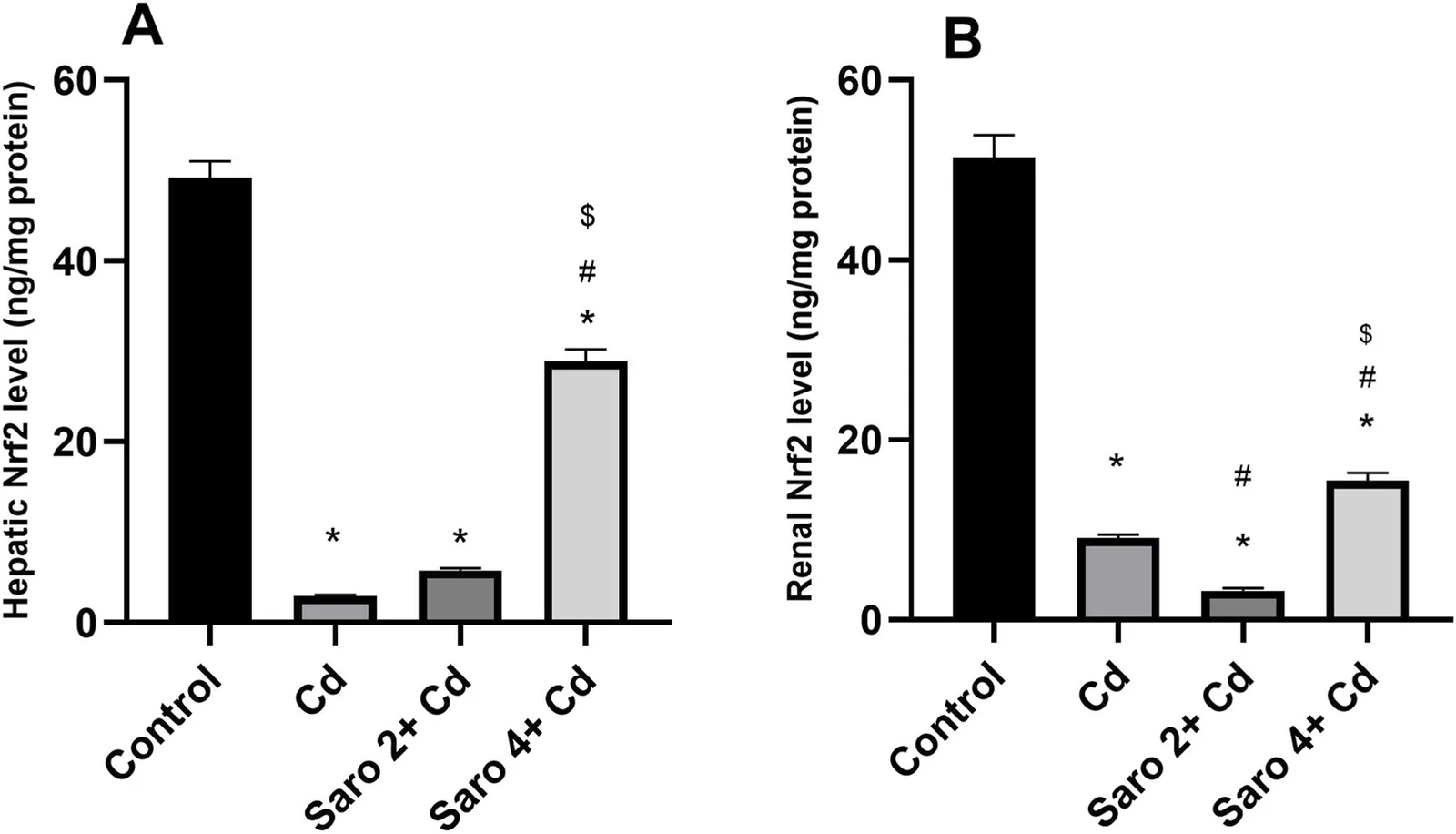
Impact of saroglitazar (2 and 4 mg/kg) on hepatic and renal Nrf2 in Cd-treated rats. **(A)** Hepatic Nrf2. **(B)** Renal Nrf2. Values are expressed as the mean ± SE (n = 6–8/group). *, #, and $ significant from control, Cd, and Saro 2+ Cd groups, respectively (*p* < 0.05), using ANOVA followed by the Tukey–Kramer test. Cd, cadmium; Nrf2, nuclear factor erythroid 2-related factor 2.

### 3.5 Impact of saroglitazar (2 and 4 mg/kg) on inflammatory markers

Concerning NF-κB, there was a profound (*p* < 0.05) increase in hepatic levels by 8.2-fold and renal levels by 6-fold compared with the control group. Upon treatment with saroglitazar (2 and 4 mg/kg), hepatic NF-κB levels were markedly (*p* < 0.05) decreased by 42% and 56.7%, respectively, and renal NF-κB levels were significantly (*p* < 0.05) reduced by 34% and 69%, respectively, when compared with the Cd group. For hepatic NF-κB, the Saro 4+ Cd group was significantly (*p* < 0.05) different from the Saro 2+ Cd group, and there was a significant difference between the control group and both groups of saroglitazar. For renal NF-κB, the Saro 4+ Cd group is significantly (*p* < 0.05) different from the Saro 2+ Cd group, and there was a significant (*p* < 0.05) difference between the control group and the Saro 2+ Cd group (Figures 5A,B).

**FIGURE 5:**
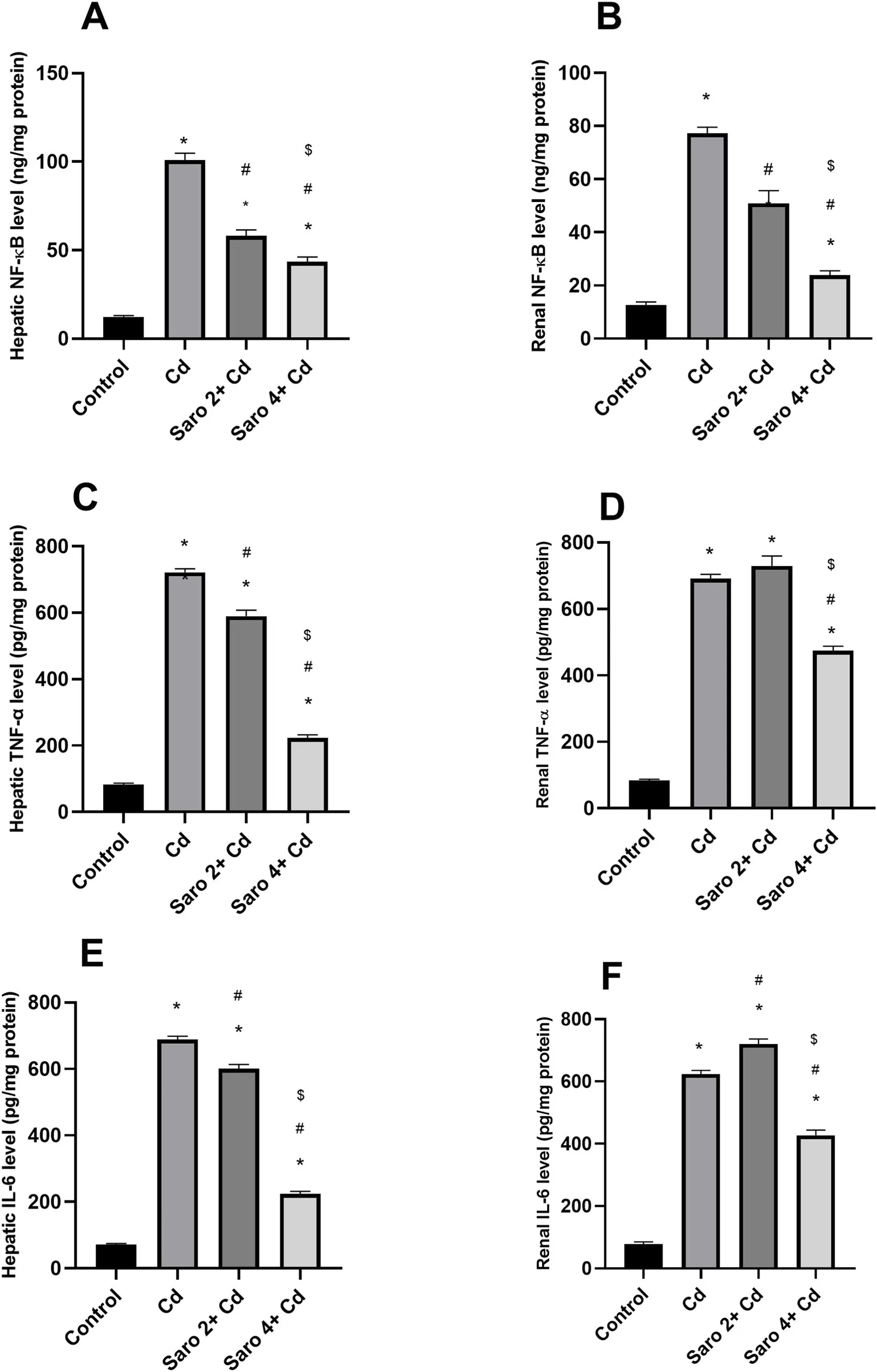
Impact of saroglitazar (2 and 4 mg/kg) on hepatic and renal inflammatory markers in Cd-treated rats. **(A)** Hepatic NF-κB. **(B)** Renal NF-κB. **(C)** Hepatic TNF-α. **(D)** Renal TNF-α. **(E)** Hepatic IL-6. **(F)** Renal IL-6. Values are expressed as the mean ± SE (n = 5–8/group). *, #, and $ significant from control, Cd, and Saro 2+ Cd groups, respectively (*p* < 0.05), using ANOVA followed by the Tukey–Kramer test. Cd, cadmium; IL-6, interleukin-6; NF-κB, nuclear factor kappa B; TNF-α, tumor necrosis factor α.

Regarding TNF-α, there was a marked (*p* < 0.05) increase in hepatic levels by 8.7-fold and renal levels by 8-fold compared with the control group. Concurrent treatment with saroglitazar (2 and 4 mg/kg) significantly (*p* < 0.05) decreased hepatic levels by 18% and 69%, respectively, and the dose 4 mg/kg decreased renal levels by 31.5% when compared with the Cd group. The Saro 4 mg/kg group is significantly (*p* < 0.05) different from the Saro 2+ Cd group. There was a significant (*p* < 0.05) difference between the control group and both groups of saroglitazar (Figures 5C,D).

About IL-6, there was a marked (*p* < 0.05) increase in hepatic levels by 9.6-fold and renal levels by 7.9-fold compared with the control group. Treatment with saroglitazar (2 and 4 mg/kg) significantly (*p* < 0.05) decreased hepatic levels by 13.5% and 31%, respectively, and the dose 4 mg/kg decreased renal levels by 31% when compared with the Cd group. The Saro 4+ Cd group is significantly different from the Saro 2+ Cd group. There was a significant (*p* < 0.05) difference between the control group and both groups of saroglitazar (Figures 5E,F).

### 3.6 Impact of saroglitazar (2 and 4 mg/kg) on liver histopathology

The photomicrographs of the liver from the control group showed the normal histological appearance of hepatic parenchyma. Liver sections from the Cd group showed steatohepatitis characterized by many intracytoplasmic vacuolation and focal areas of inflammatory aggregations that surrounded and replaced a necrotic hepatocyte. These inflammatory foci consisted of aggregations of lymphocytes, macrophages, and neutrophils replacing a hypereosinophilic cell with a pyknotic nucleus. The Saro 2+ Cd group showed a few inflammatory foci, and the rest of the hepatic parenchyma appeared normal. The Saro 4+ Cd group showed the normal histological appearance of hepatic parenchyma (Figures 6A–D). Semiquantitative analysis for hepatic necrosis, inflammation, and steatosis scores is shown in Figures 6E–G.

**FIGURE 6:**
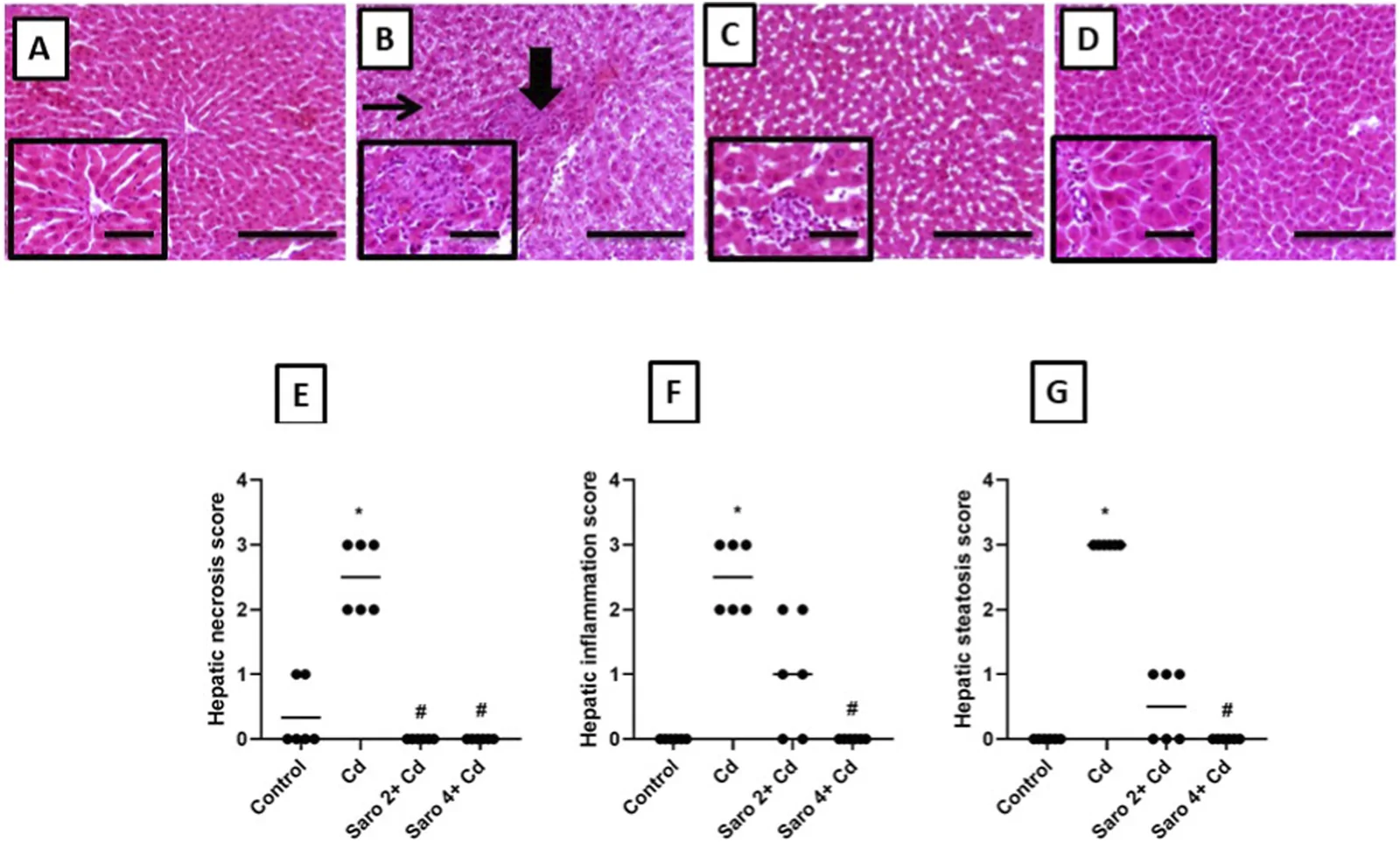
Impact of saroglitazar (2 and 4 mg/kg) on hepatic histopathology in Cd-treated rats. **(A)** Control group showing normal histological appearance of hepatic parenchyma, inset higher power on normal hepatocytes. **(B)** Cd group showing steatohepatitis characterized by many intracytoplasmic vacuolation (thin arrow) and focal areas of inflammatory aggregations that surround and replace a necrotic hepatocytes (thick arrow); inset: these inflammatory foci consisted of aggregations of lymphocytes, macrophages, and neutrophils replacing a hypereosinophilic with a pyknotic nucleus (necrosis). **(C)** Saro 2+ Cd group showing a few inflammatory foci (thick arrow), and the rest of the hepatic parenchyma appeared normal; see inset inflammatory foci. **(D)** Saro 4+ Cd group showing normal histological appearance of hepatic parenchyma. **(E–G)** Semiquantitative quantitative analysis of hepatic necrosis, inflammation, and steatosis scores, respectively. Data are expressed as the median ± interquartile range (n = 6). * and # significant from control and Cd groups, respectively (*p* < 0.05), using the Kruskal–Wallis test followed by Dunn’s multiple comparison *post hoc* test.

### 3.7 Impact of saroglitazar (2 and 4 mg/kg) on kidney histopathology

The photomicrograph of the kidney from the control group showed normal renal parenchyma and normal glomerular and tubular architecture. Renal sections from the Cd group showed extensive interstitial fibrosis, dense fibrous connective tissue, inflammatory cells, interstitial congestion and haemorrhage. The Saro 2+ Cd group showed focal minimal to mild interstitial inflammation. The Saro 4+ Cd group showed the normal architecture of renal parenchyma except for a few interstitial congestions (Figures 7A–D). Semiquantitative scores for renal necrosis, inflammation, congestion, and fibrosis are shown in Figures 7E–H.

**FIGURE 7:**
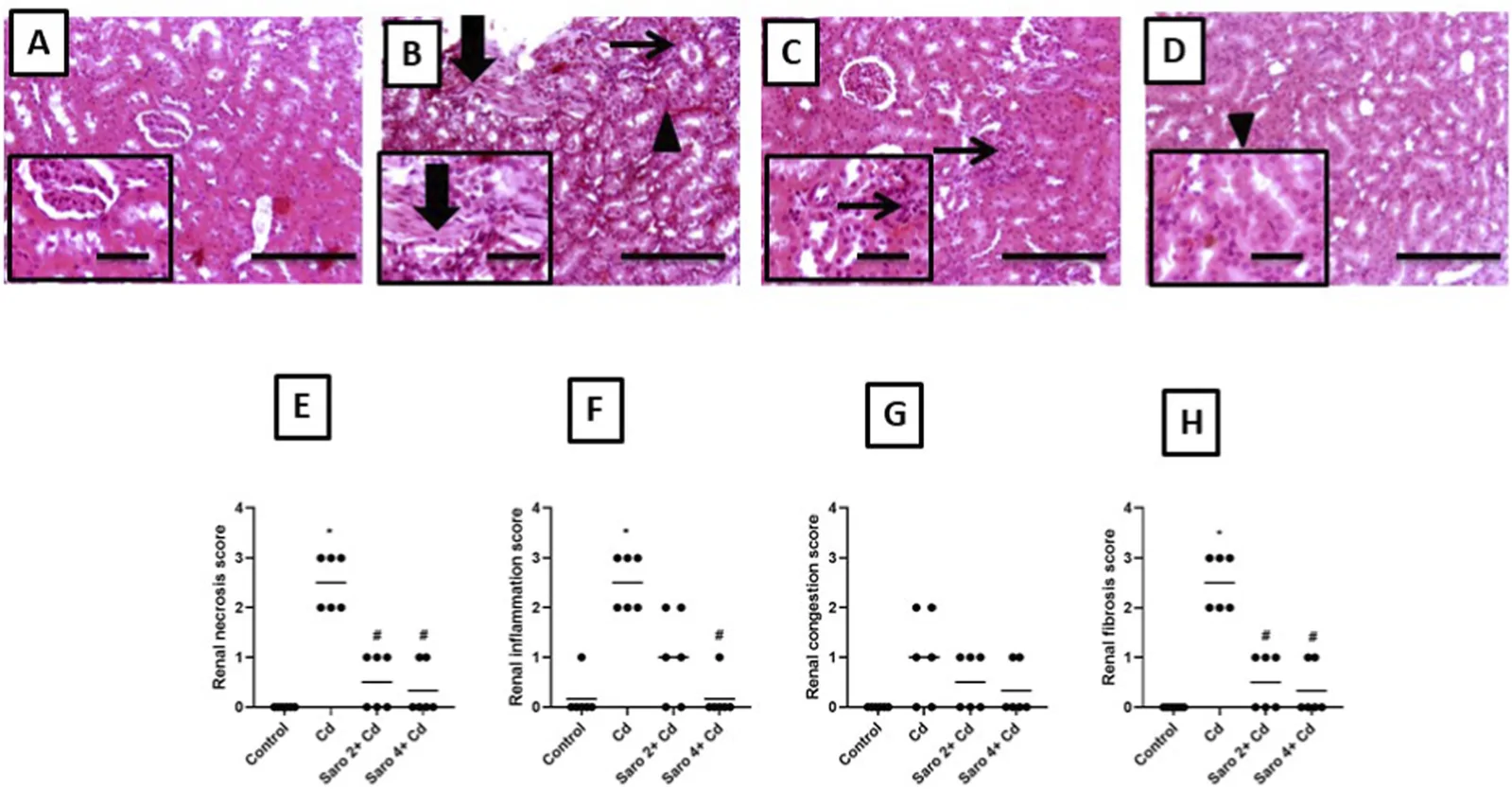
Impact of saroglitazar (2 and 4 mg/kg) on renal histopathology in Cd-treated rats. **(A)** Control group showing normal renal parenchyma; inset: normal glomerular and tubular architecture. **(B)** The Cd group showing severe interstitial fibrosis (thick arrow) admixed with inflammatory cells (thin arrow), replacing the normal architecture with interstitial congestion (arrowhead); inset: dense fibrous connective tissue (thick arrow) replacing a necrotic tubule and admixed with hemorrhage. **(C)** Saro 2+ Cd group showing focal minimal to mild interstitial inflammation (thin arrows); see inset. **(D)** Saro 4 + Cd group showing normal architecture of the renal parenchyma except for a few interstitial congestions (arrowhead); see inset. **(E–H)** Semiquantitative quantitative analysis of renal necrosis, inflammation, congestion, and fibrosis scores, respectively. Data are expressed as the median ± interquartile range (n = 6). * and # significant from control and Cd groups, respectively (*p* < 0.05), using the Kruskal–Wallis test followed by Dunn’s multiple comparison *post hoc* test.

### 3.8 Impact of saroglitazar (2 and 4 mg/kg) on immunohistochemical analysis

The representative photographs of IHC of the control group showed negative immunostaining expression of caspase-3 in hepatocytes. Sections from the Cd group showed moderate to high cytoplasmic and nuclear expression of caspase-3 in hepatocytes. The Saro 2+ Cd group showed mild scattered immunopositive-stained hepatocytes. The Saro 4+ Cd group showed few focal immunopositive-stained hepatocytes (Figures 8A–H).

**FIGURE 8:**
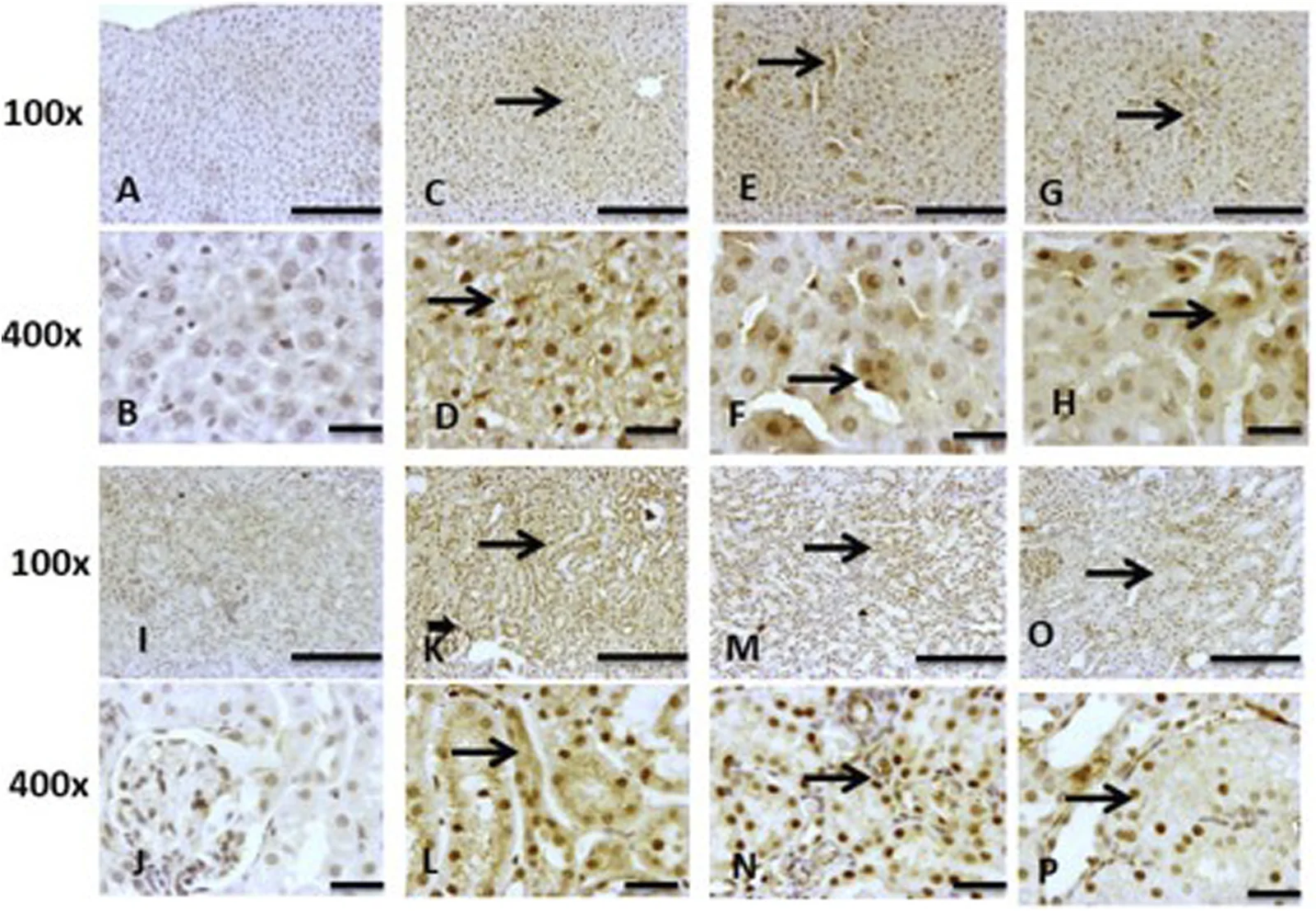
Impact of saroglitazar (2 and 4 mg/kg) on immunohistochemical analysis of hepatic and renal caspase-3 expression. **(A, B)** Control group showing negative immunostaining expression of caspase in hepatocytes. **(C, D)** Cd group showing moderate to high cytoplasmic and nuclear expression of caspase-3 in hepatocytes. **(E, F)** Saro 2+ Cd group showing mild scattered immunopositive-stained hepatocytes. **(G, H)** Saro 4+ Cd group showing a few focal immunopositive-stained hepatocytes. **(I, J)** The control group showing negative to a few immunostained glomeruli and renal tubular epithelial cells. **(K, L)** Cd group showing diffuse, highly immunopositive-stained renal tubules and glomeruli. **(M, N)** Saro 2+ Cd group showing negative to mild nuclear immunopositive-stained renal tubules. **(O, P)** Saro 4+ Cd group showing a few immunopositive-stained renal tubules. Thin arrow = positive renal tubules, thick arrow = positive glomeruli. Image magnification **(A, C, E, G, I, K, M, O)** 100×, scale = 100 μm; **(B, D, F, H, J, L, N, P)** 400×, scale = 50 µm.

The representative photomicrograph of the kidney from the control group showed negative to a few immunostained glomeruli and renal tubular epithelial cells. Sections from the Cd group showed diffuse, highly immunopositive-stained renal tubules and glomeruli. The Saro 2+ Cd group showed negative to mild nuclear immunopositive-stained renal tubules. The Saro 4+ Cd group showed a few immunopositive-stained renal tubules (Figures 8I–P).

Figures 9A,B show the semiquantitative analysis of caspase immunostaining in hepatic and renal tissues expressed as % of immunopositive cells.

**FIGURE 9:**
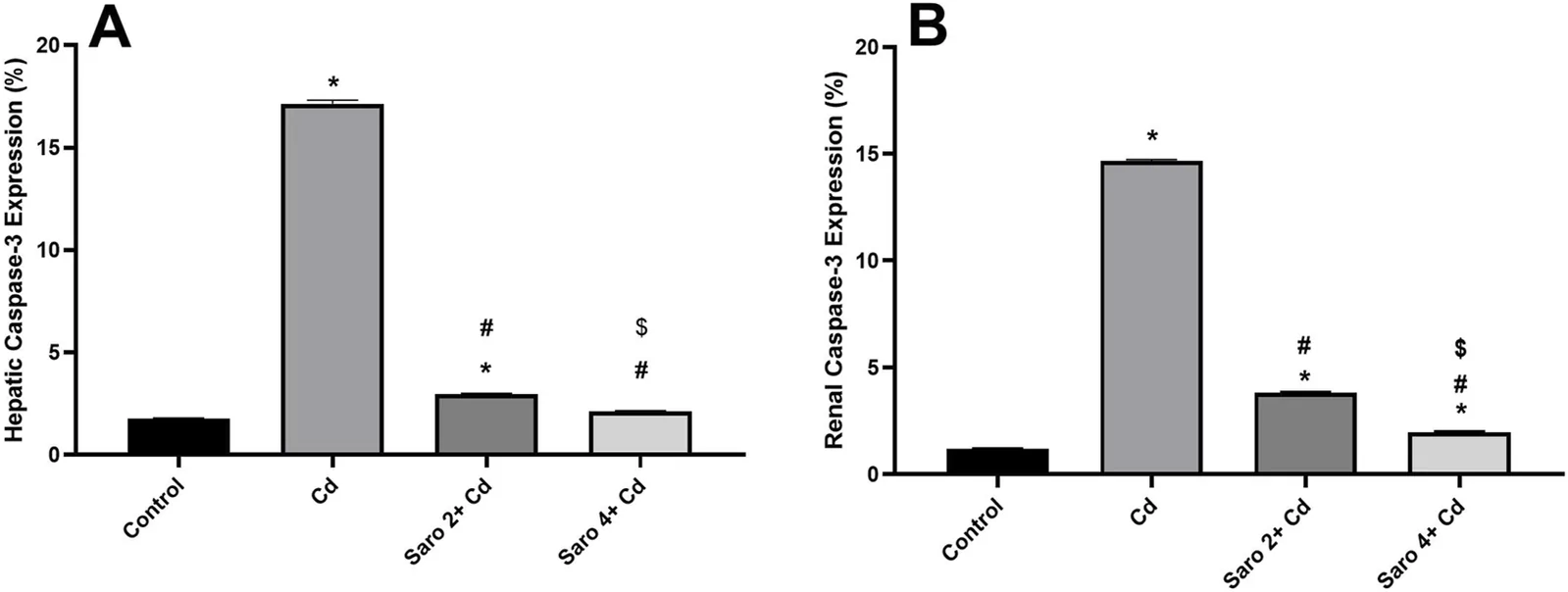
Impact of saroglitazar (2 and 4 mg/kg) on the percentage of immunopositive cells for caspase-3. Semiquantitative analysis of caspase-3 immunostaining in **(A)** hepatic and **(B)** renal tissues expressed as % of immunopositive cells. Values are expressed as the mean ± SE (n = 6/group). *, #, and $ significant from control, Cd, and Saro 2+ Cd groups, respectively (*p* < 0.05), using ANOVA followed by the Tukey–Kramer test.

Representative negative control hepatic sections (low and its corresponding high-power view) showed negatively stained hepatic sections without any caspase-3 expression (Figures 10A–F). Representative negative control renal sections (low-power and its corresponding high-power view) showed negatively stained renal sections without any caspase-3 expression (Figures 10G–L).

**FIGURE 10:**
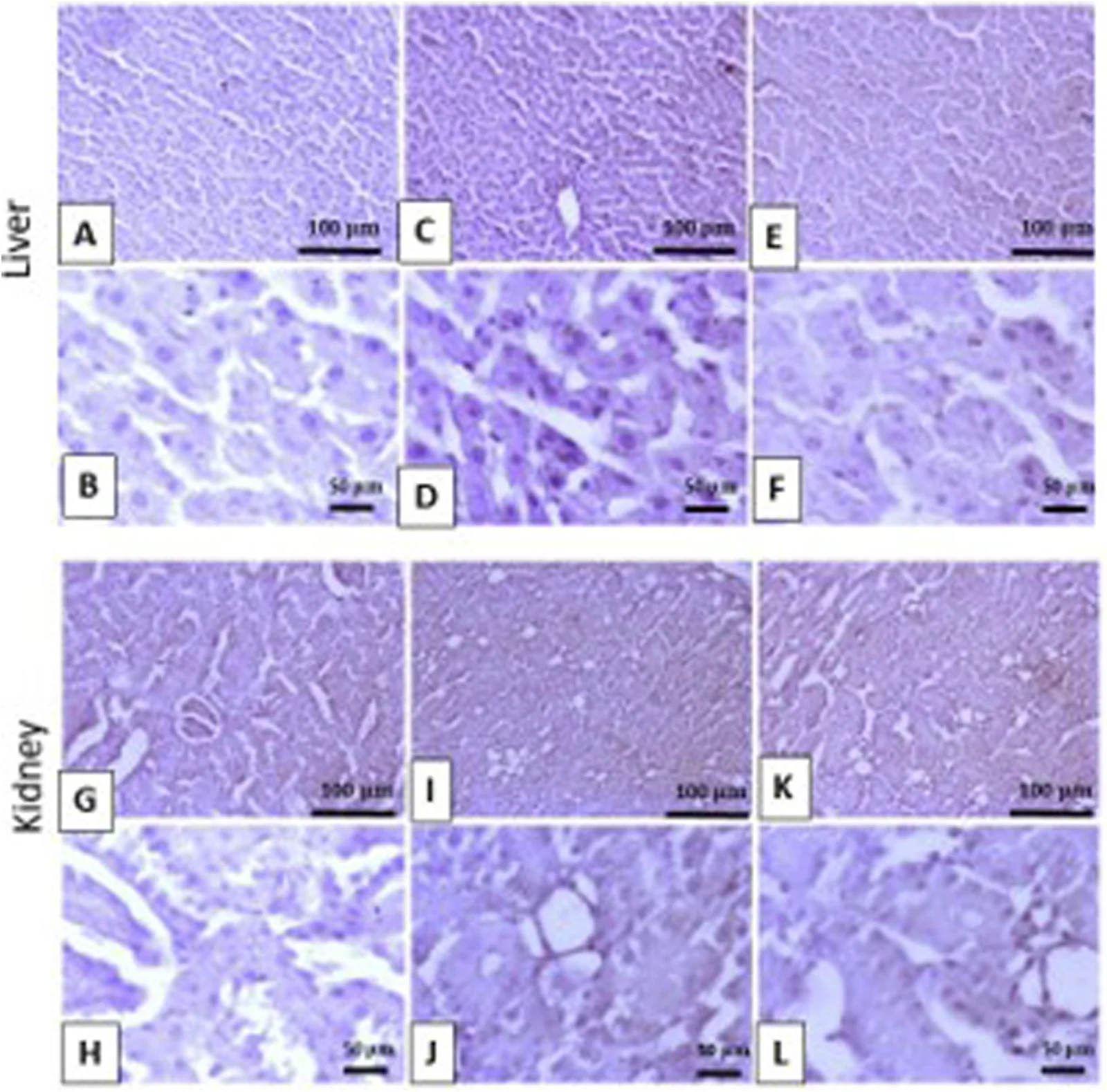
Impact of saroglitazar (2 and 4 mg/kg) on negative controls of caspase-3. **(A–F)** Representative hepatic sections showing negatively stained hepatic sections without any caspase-3 expression. **(G–L)** Representative negative control renal sections showing negatively stained renal sections without any caspase-3 expression. Image magnification = 100×, bar = 100 μm; 400×, bar = 50 μm.

## 4 Discussion

Humans and animals may suffer detrimental effects from Cd, which is considered a naturally hazardous metal. Exposure to Cd results in its buildup mostly in metabolically active organs, such as the liver and kidneys (Omar and El-Sayed, 2024). In this study, the effects of Cd poisoning on the liver and kidneys were investigated, along with the possible impacts of how saroglitazar could lessen these dreadful effects. Histological and biochemical evaluations were applied to determine hepatic and renal damage resulting from Cd exposure and investigate the possible protective effects of saroglitazar and the fundamental mechanisms behind these effects. The liver is the main organ responsible for drug metabolism and is regarded as the primary site for protein synthesis; consequently, cellular damage caused by Cd can be assessed by evaluating hepatic enzyme levels in serum, comprising AST, ALT, and ALP, in addition to albumin levels (Chiang, 2014). Thus, when the liver is hurt by necrosis, inflammation, or a blocked bile duct, liver enzymes are released into the serum. These enzymes give important physiological information about the health state (Farag and Alagawany, 2023). The findings of the current investigation indicated that Cd significantly elevated serum AST, ALT, ALP, GGT, and bilirubin, besides reduced albumin levels relative to the control group. These findings are substantiated by data published by other researchers (Renugadevi and Prabu, 2010; El-Demerdash et al., 2004; Toppo et al., 2015), which reported that levels of albumin in rats exposed to Cd decreased, indicating a reduced liver’s ability to produce proteins. Also, it has been stated that elevated levels of ALP and GGT serve as crucial indicators for detecting liver damage (Kumar et al., 2021), and increased serum bilirubin concentrations are a definitive indicator of hepatic injury (Mladenović et al., 2014). Moreover, it has been documented that elevated serum total and direct bilirubin, a definitive marker of hepatic injury, may result from heightened hepatocyte damage, inflammatory cell infiltration, and biliary tract dysfunction, which impede hepatocyte bilirubin absorption and lead to the accumulation of lipid droplets in Cd-treated rats (Elgharib and Abdelhamid, 2025).

In contrast, oral administration of saroglitazar in both doses markedly decreased the serum levels of AST, ALT, and ALP when compared with the Cd group. These observations agreed with an earlier study which reported that saroglitazar exerted a hepatoprotective effect against 5-fluorouracil (Alharbi et al., 2024).

Surprisingly, a non-monotonic dose–response was observed for ALP, GGT, and bilirubin, where 2 mg/kg produced greater improvement than 4 mg/kg. Such paradoxical responses reported with PPAR-α/γ agonists may be attributed to receptor desensitization, off-target interactions at higher doses, or bell-shaped pharmacodynamic behavior. These findings warrant further investigation.

The biochemical results of this investigation indicated renal impairment in Cd-exposed rats as evidenced by a substantial increase in the serum levels of creatinine and urea. These observations agreed with an earlier study that reported an increment in the serum levels of creatinine and urea following Cd exposure (Ashour, 2014). In contrast, such impairment was not seen in saroglitazar-treated animals, in which there was a marked decrease in the serum levels of creatinine and urea. These observations were in line with an earlier study which reported that saroglitazar ameliorated 5-fluorouracil-induced renal damage in rats (Alharbi et al., 2024).

Cd entry into the cytoplasm may induce the production of ROS, lipid peroxidation, glutathione depletion, protein cross-linking, and inflammation. As a result, proinflammatory cytokines increase, causing the death of renal cells and consequent renal dysfunction (Ansari et al., 2017). The results of the present study indicated that GSH and SOD enzyme activity significantly decreased; however, MDA levels were increased, indicating enhanced lipid peroxidation. Conversely, administration of saroglitazar restored oxidant/antioxidant balance as manifested by elevated GSH and SOD concomitant with marked reduction in MDA. These observations are consistent with an earlier study which reported that saroglitazar attenuated oxidative stress in a UUO model in rats (Makled and El-Kashef, 2020).

Nrf2 is a transcription factor that controls the expression of several cytoprotective genes in reaction to electrophilic and oxidative stress. Normally, Kelch-like ECH-associating protein 1 (Keap1) sequesters Nrf2, causing it to exist in the cytoplasm in an inactive form. According to Klaassen and Reisman (2010), Nrf2 is liberated from Keap1 under oxidative stress conditions and moves into the nucleus, where it triggers the production of cytoprotective genes including HO-1. In this study, Nrf2 has been assessed only at the total protein level using ELISA. Our results showed that Cd substantially reduced Nrf2 levels when compared with the control group. By contrast, saroglitazar effectively elevated Nrf2 levels when compared with the Cd group. This may contribute to the hepatorenal protective effects of saroglitazar. PPAR-γ activation has previously shown an antioxidative effect in an intracerebral hemorrhage model in rats, which could be mediated via the activation of the Nrf2 pathway (Hang et al., 2020). Also, it has been reported that Nrf2 and PPAR-γ activators have collaborative power against metabolic and drug-induced oxidative injury (Lee, 2017).

Oxidative stress may activate NF-κB, which stimulates the synthesis of proinflammatory cytokines such as TNF-α and IL-6 (Makled and El-Kashef, 2020). NF-κB is one of the fast-acting transcription factors in humans and a regulator of the innate type of immunity, and it serves as a mediator between inflammation and oxidative damage. NF-κB is normally inactivated in the cytoplasm by association with its inhibitory protein IκB. Oxidative stress induces IκB phosphorylation by the IκB kinase (IKK) complex. This phosphorylation promotes IκB degradation, leading to the release of NF-κB dimers, which translocate into the nucleus and stimulate downstream target genes (Li et al., 2021). TNF-α is a cytokine that mediates cell signaling and has a role in systemic inflammation by initiating the acute phase response (Omar and El-Sayed, 2024). In this study, exposure to Cd significantly increased the hepatic and renal levels of NF-κB, TNF-α, and IL-6 relative to the control group. These observations are in agreement with an earlier study which reported that Cd has a significant influence on the concentrations of TNF-α and IL-6 (Omar and El-Sayed, 2024). In contrast, saroglitazar markedly decreased the hepatic and renal levels of NF-κB, TNF-α, and IL-6 compared with the Cd group. These findings agreed with a former study which reported that saroglitazar ameliorated 5-fluorouracil-induced inflammation in rats (Alharbi et al., 2024). Additionally, PPARs have been previously suggested to exert anti-inflammatory activities by antagonizing the NF-κB pathway in macrophages in ischemia-reperfusion lung injury (Zhu et al., 2016).

According to earlier research, apoptosis plays a significant role in the pathophysiology of intestinal integrity and barrier function disruption, which can lead to an inflammatory cascade. In this investigation, apoptosis was assessed using caspase-3 immunohistochemistry only; Cd increased the percentage of immunopositive cells for caspase-3. This observation is consistent with a prior study that found Cd-induced apoptosis of the kidney and increased protein expression levels of caspase-3 (Huang et al., 2022). This action may be explained by Cd capacity to raise TNF-α, a potent apoptosis inducer, and specifically caspase-3 activation. Saroglitazar pretreatment, however, effectively reverses this effect. Saroglitazar has been demonstrated to decrease pyroptosis after LPS-induced hepatic and renal injury in rats (Francis et al., 2023).

To sum up, saroglitazar may help prevent Cd-induced hepatic and renal damage in rats by reducing oxidative stress, inflammatory response, and apoptosis through the modulation of NF-κB and Nrf2 pathways. Saroglitazar increased Nrf2 protein levels; however, confirmation of Nrf2 pathway activation requires further mechanistic studies.

## 5 Limitations

This study has some limitations such as the absence of a chronic model, use of only one species and sex, use of a prophylactic protocol instead of a therapeutic protocol, and lack of a saroglitazar-only group (a group receiving saroglitazar only without Cd), which would be useful in determining whether the drug itself induces any biochemical or histopathological changes. Another limitation is that pathway modulation was inferred from measuring protein levels only, and further mechanistic studies are required. Furthermore, Cd concentrations were not measured; this is considered a limitation because we are unable to distinguish whether saroglitazar reduced Cd accumulation (pharmacokinetic effect) or acted downstream (pharmacodynamic protection). A positive control group was not included, which limits benchmarking of saroglitazar’s efficacy.

## Data Availability

The raw data supporting the conclusions of this article will be made available by the authors, without undue reservation.
